# Río Tinto: A Geochemical and Mineralogical Terrestrial Analogue of Mars

**DOI:** 10.3390/life4030511

**Published:** 2014-09-15

**Authors:** Ricardo Amils, David Fernández-Remolar, the IPBSL Team

**Affiliations:** 1 Centro de Biología Molecular Severo Ochoa (CSIC-UAM), Universidad Autónoma de Madrid, Cantoblanco, 28049 Madrid, Spain; 2 Centro de Astrobiología (CSIC-INTA), km 4 carrtera Ajalvir, 28850 Torrejón de Ardoz, Spain; E-Mail: kernnunnos@gmail.com

**Keywords:** acidophiles, Río Tinto, Iberian Pyrite Belt, metal sulfides, iron oxidation, iron cycle, sulfur cycle, iron minerals, jarosite, Mars

## Abstract

The geomicrobiological characterization of the water column and sediments of Río Tinto (Huelva, Southwestern Spain) have proven the importance of the iron and the sulfur cycles, not only in generating the extreme conditions of the habitat (low pH, high concentration of toxic heavy metals), but also in maintaining the high level of microbial diversity detected in the basin. It has been proven that the extreme acidic conditions of Río Tinto basin are not the product of 5000 years of mining activity in the area, but the consequence of an active underground bioreactor that obtains its energy from the massive sulfidic minerals existing in the Iberian Pyrite Belt. Two drilling projects, MARTE (Mars Astrobiology Research and Technology Experiment) (2003–2006) and IPBSL (Iberian Pyrite Belt Subsurface Life Detection) (2011–2015), were developed and carried out to provide evidence of subsurface microbial activity and the potential resources that support these activities. The reduced substrates and the oxidants that drive the system appear to come from the rock matrix. These resources need only groundwater to launch diverse microbial metabolisms. The similarities between the vast sulfate and iron oxide deposits on Mars and the main sulfide bioleaching products found in the Tinto basin have given Río Tinto the status of a geochemical and mineralogical Mars terrestrial analogue.

## 1. Introduction

The NASA Astrobiology roadmap [1] highlights the interest in extreme environments and the microorganisms that live in them in evaluating the possible existence of life beyond Earth. Acidophiles are of special interest, because the environments in which they thrive are the product of the chemolithotrophic metabolism of microorganisms that obtain energy from reduced mineral substrates and are not adaptations to geophysical constraints, as with most extremophiles. The meager requirements they have places them among the best candidates for a successful primitive energy conservation system.

The Viking mission, considered the first astrobiological mission devoted to the search for signs of life on Mars, concluded that life had little chance of developing there given the extreme conditions detected on its surface [2]. In the last forty years, important advances in microbiology, mainly in the characterization of extreme environments, have challenged this pessimistic point of view. Thanks to research on extremophiles, we now know that life is extremely robust and adapts rapidly to different conditions.

Natural acidic environments have two major origins. One associated with volcanic activities, the sulfur world, and the other linked to mining activities. Coal and metal mining operations expose sulfidic minerals to the action of aerobic chemolithotrophic microorganisms, facilitating their growth and generating acid mine drainage (AMD) or acid rock drainage (ARD), which are the cause of important environmental problems [3].

The mechanism by which microbes obtain energy by oxidizing sulfide mineral has been controversial for many years [4], but the demonstration that ferric iron present in the cell envelopes of leaching microorganisms is responsible for the electron transfer from insoluble mineral substrates to the microbial electron transport chain has clarified the situation [5]. The differences observed by using various sulfide minerals are determined by chemical oxidation mechanisms, which depend on the crystallographic structure of the mineral substrates [6].

The acidophilic strict chemolithotroph, *Acidithiobacillus ferrooxidans* (formerly *Thiobacillus ferrooxidans*), was first isolated from a coal mine in the 1940s [7]. Although *At. ferrooxidans* can obtain energy b oxidizing either reduced sulfur compounds or ferrous iron, bioenergetic considerations gave more prominence to the sulfide oxidation reaction [8,9]. The discovery that some strict chemolithotrophs, such as *Leptospirillum ferrooxidans*, thrive using ferrous iron as their only source of energy and that their role in bioleaching operations and the generation of AMDs is much more important than previously thought has completely changed this point of view [10]. Furthermore, it is well established that iron can be oxidized not only aerobically, but anaerobically, coupled to anoxygenic photosynthesis, using ferrous iron as environmental reducing power or anaerobic respiration using nitrate as an electron acceptor (denitrification) [11,12].

The recent discovery of subsurface chemolithotrophic microorganisms participating in a radiation-free biosphere has opened an interesting perspective in astrobiology [13,14,15,16]. There is a growing list of alternative sources of lithotrophic substrates (Fe^2+^, S^2−^, S^0^, As^3+^, Mn^2+^, *etc.*), which widens the range of metabolic versatility of this energy conservation system. Furthermore, sulfur- and iron-oxidizing microorganisms coupled to sulfur- and iron-reducers have a critical role in the maintenance of the sulfur and iron cycles, two fundamental biogeochemical cycles.

Acidic environments vary greatly in their physicochemical characteristics and microbial ecology. High temperatures may be the result of biological activity, facilitating colonization by thermotolerant and thermophilic acidophiles. Acidic ecosystems associated with mining activities are, at the geological scale, very young. However, some metal mines have a rather long history. Mines such as Río Tinto are known to have been in operation more than 5000 years ago [17].

## 2. Rio Tinto 

**Figure 1:**
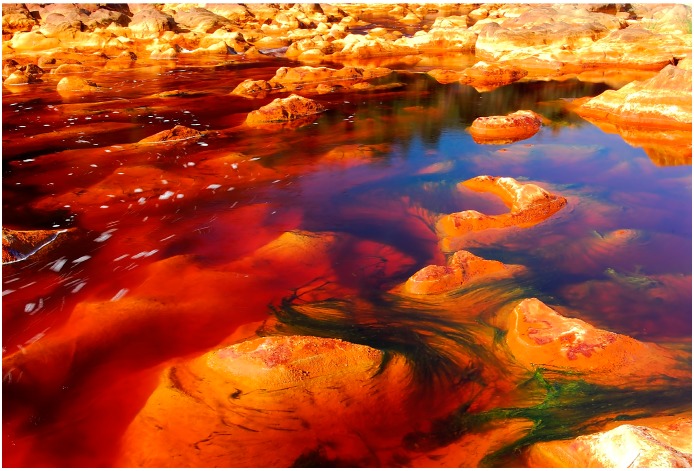
Río Tinto basin at Berrocal (J. Segura).

Río Tinto (Figure 1) is an unusual ecosystem due to its acidity (mean pH 2.3), size (92 km long), high concentration of heavy metals (Fe, Cu, Zn, As, *etc.*) and unexpectedly high level of microbial diversity [18,19,20]. Río Tinto springs up in Peña de Hierro, in the core of the Iberian Pyrite Belt (IPB), and flows into the Atlantic Ocean at Huelva. The IPB is one of the largest sulfidic deposits in the world. Massive bodies of iron and copper sulfides, as well as minor quantities of lead and zinc sulfides constitute the main mineral ores. Its formation by hydrothermalism took place during the Hercynian orogenesis [21,22,23].

The basin of the river covers an area of 1700 km^2^. The measured redox potentials in the Tinto basin range from +280 to +650 mV, and the oxygen content varies from saturation to complete anoxic conditions. The Tinto basin exhibits a Mediterranean climate. An important characteristic of the Tinto ecosystem is its constant acidic pH, which is a direct consequence of the strong buffer capacity of ferric iron. Due to its size and easy access, Río Tinto is considered an excellent model for the study of the microbial ecology of extreme acidic environments.

The combined use of conventional and molecular microbial ecology methodologies has led to the identification of the most representative Tinto basin prokaryotic microorganisms [18,19,20,24,25,26]. Eighty percent of the water column diversity corresponds to microorganisms belonging to three bacterial genera: *Acidithiobacillus*, *Leptospirillum* and *Acidiphilium*, all members of the iron cycle [20]. *At. ferrooxidans* can oxidize ferrous iron aerobically and reduce ferric iron in anaerobic conditions [27,28]. All *Leptospirillum* isolated from Río Tinto are aerobic iron oxidizers. All *Acidiphilium* isolates can oxidize organic compounds using ferric iron as an electron acceptor (anaerobic respiration). Some *Acidiphilium* isolates can use ferric iron as an electron acceptor in the presence of oxygen [29,30]. Although other iron-oxidizers (*Ferrovum* spp., *Ferrimicrobium* spp., *Ferroplasma* spp. and *Thermoplasma acidophilum*) or iron-reducers (*Ferrimicrobium* spp., *Acidisphaera* spp., *Metallibacterium* spp. and *Acidobacterium* spp.) have been identified in the Tinto basin [20,25,26], their low numbers detected by *in situ* fluorescence hybridization suggest that they play a minor role in the operation of the iron cycle, at least in the water column.

Concerning the sulfur cycle, only *At. ferrooxidans* is found in significant numbers in the water column. This bacterium can oxidize both ferrous iron and reduced sulfur compounds. Reduced sulfur compounds can be oxidized aerobically and anaerobically. Sulfate reducing microorganisms that close the sulfur cycle have been detected in the sediments in various locations along the river [26,28,31,32,33].

Due to the biotechnological interests of the aerobic iron oxidizing microorganisms, interest in the characterization of the anoxic sediments from acidic environments has been overshadowed until recently, with few exceptions [34]. Recently, a comparative analysis of the sediments and the water column of different samples along the physicochemical gradient of Río Tinto have been performed [26]. This study showed a significantly higher level of biodiversity in the anaerobic sediments when compared to their water column counterparts. Nearly all of the microorganisms identified in this study were, in one way or another, related to the iron cycle. Most had been previously detected and/or isolated in AMD sites [3,20,25,26,35,36] or at biohydrometallurgical operations [37]. Nonetheless, some bacteria, such as members of Actinobacteria, Firmicutes, Acidobacteria, Planctomycetes and Chloroflexi, have been identified recently in the Tinto basin [26].

An in-depth analysis of two anoxic sediments from the Tinto basin has shown major phylogenetic differences among sample sites [31]. In one of the sediments, JL Dam, the most numerous group of bacteria corresponded to the phylum, Firmicutes (56.6%), followed by the phylum, Acidobacteria (27.3%), and the class, Deltaproteobacteria (11.6%). In the SN Dam, Proteobacteria was the most represented phylum (72.1%), followed by Actinobacteria (20.4%), while organisms of the Firmicutes and Acidobacteria phyla were present in low percentages. In the strict anoxic conditions detected in the lower part of the sediments of the Tinto basin, sulfate reduction is a recurrent microbial activity, a consequence of the high concentration of sulfates existing in the system.

It is usually assumed that the toxicity of high metal concentrations in acidic habitats limits eukaryotic growth and diversity. However, colorful biofilms cover large surfaces of the Tinto basin. In fact, it has been observed that eukaryotic microorganisms contribute over 60% of the Tinto basin biomass [18]. A significant number of eukaryotic species thriving in Río Tinto are photosynthetic. Among them, chlorophytes related to different genera, such as *Chlamydomonas*, *Dunaliella* and *Chlorella*, are the most abundant eukaryotic microorganisms in the river [19,38,39,40]. Filamentous algae, represented by the genera, *Zygnemopsis* and *Klebsormidium*, have been also found. The most extreme part of the river is inhabited by a eukaryotic community dominated by two species related to the genera, *Dunaliella* and *Cyanidium*. Pennate diatoms are also present in the river, forming large brown biofilms [38,39]. Photoautotrophic flagellates of the genera, *Euglena*, *Bodo* and *Ochromonas*, are also widely distributed along the river. The dominant ciliate taxa belong to the order *Hypotrichida*. Amoebas are frequently found feeding on large diatoms, even in the most acidic part of the river. Heliozoa seem to be the characteristic top predators of the benthic food chain [19,38,39]. The only animal found in the river is a species of bdelloid rotifer related to the genus, *Rotifera* [19].

Among decomposers, fungi are the most diverse, and both unicellular and filamentous forms are present [18,41]. A recent characterization of Río Tinto basin samples from different stations along the river have rendered more than three hundred and fifty fungal isolates, which have been identified by ITS region sequence analysis. This analysis revealed Ascomycetes as the most abundant phylum, while Basidiomycetes and Zygomycetes accounted for less than 2% of the sequenced isolates. Of the Ascomycetes, 52% clustered within the Eurotiomycetes class, while 27% grouped with the Dothideomycetes and 17% with the Sordariomycetes. Concerning metal tolerance, Eurotiomycetes and Sordariomycetes isolates showed, in general, a high level of resistance to toxic heavy metals, much higher than the concentrations detected in the river, while members of the Dothideomycetes showed a level of resistance to concentrations similar to those detected in the water column.

However, not only unicellular eukaryotic systems develop in the extreme conditions of Río Tinto. Different plants can be found growing in the acidic soils of the Tinto basin [42,43,44]. The strategies used by these plants to overcome the physiological problems associated with the extreme conditions of the habitat are diverse. Some are resistant to the high concentration of heavy metals present in the soils in which they grow. Others specifically concentrate metals in different plant tissues. The analysis of the iron minerals found in the rhizomes and leaves of *Imperata cylindrica*, an iron hyperaccumulator perennial grass growing in the Tinto basin, showed significant concentrations of jarosite and iron hydroxides [42,45,46]. These results suggest that the management of heavy metals, in general, and iron, in particular, is much more complex in plants than what has been described to date. Furthermore, these results prove that multicellular complex systems can also develop in some extreme conditions, like those existing in Río Tinto.

## 3. Iron Bioformations in the Tinto Basin

**Figure 2:**
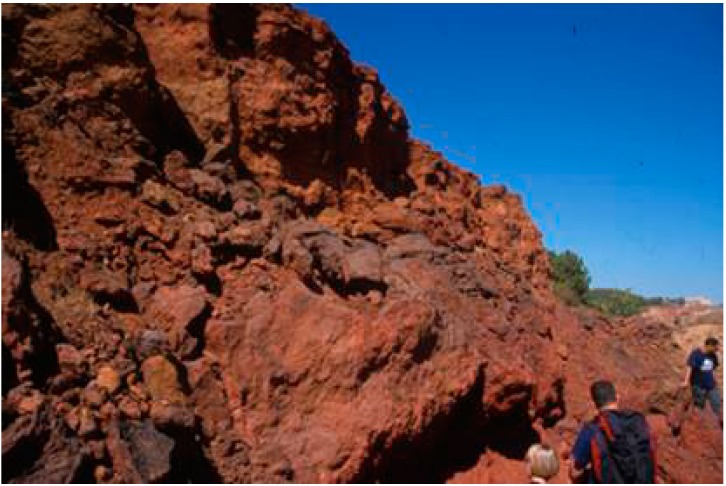
Alto de la Mesa old terrace.

Most of the Tinto basin biomass is located on the surface of the rocks in the riverbed. It is made up of dense biofilms, composed mainly of filamentous algae and fungi in which prokaryotic microorganisms are trapped. Significant iron mineral precipitation occurs on the negatively-charged surface of these biofilms, generating iron precipitates, which grow following the hydrological cycles and consolidate as iron-rich deposits elevated above the present river in the form of fluvial terraces (Figure 2) [47,48].

Seasonal evaporation of river water drives precipitation of hydronium jarosite and schwertmannite, while copiapite, coquimbite, gypsum and other sulfate minerals generate efflorescence brought to the surface by capillary action [48]. During the wet season, hydrolysis of sulfate salts added to the effect of iron hydrolysis, facilitating the precipitation of amorphous iron oxyhydroxides.

The oldest terraces show increasing goethite crystallinity and its replacement by hematite over time. Organic matter does not preserve well in the Río Tinto sediments, but biosignatures imparted to sedimentary rocks as macroscopic textures of coated microbial streamers, surface blisters originating from biogenic gas and microfossils preserved in iron oxides can help to shape strategies for their detection in extant or future space exploration missions [48,49,50]. Interestingly, the specific biomineralization of hydronium jarosite by a filamentous fungus isolated from the Tinto basin, *Purpureocillium lilacinum*, in non-permissive ionic conditions has been recently described [51]. Furthermore, the presence of siderite (FeCO_3_) in the modern sediments of the river [52], which has also been generated in cultures of the acidophilic heterotrophic iron reducer, *Acidiphilium* sp., in acidic conditions [53], strongly suggests that the presence of biological nucleation sites can modify the expected mineral precipitation schemes offered by the bulk physicochemical conditions in which microorganisms grow.

The recent detection of protein fragments and other organic molecules [54,55] in the ancient terraces of the Rio Tinto basin evidences that its acidic and ferruginous environment promotes the preservation of molecules bearing information about the producing organism that inhabited this extreme environment over the last million years.

Until recently, it was generally accepted that the extreme conditions found in Río Tinto were the direct result of the mining activities performed in the area during the last 5000 years [17,56]. New geological, geophysical and hydrogeological information supports the hypothesis that this is not the case. The location of the recharge area of the Peña de Hierro aquifer was recently determined northwest of the pit lake at a depth ranging from −100 to −400 m [57]. The groundwater moves southwards along the fracture network, and when it reaches the remnants of massive and/or stockwork sulfide bodies, located at −500 m in depth, the water interacts with the mineral substrate, facilitating the metabolism of chemolithotrophic microorganisms and generating acidic fluids [57,58]. Groundwater is eventually pumped along open strike-slip normal faults to reach the surface, where it sources the acidic springs that feed the headwaters of Río Tinto. The generation of acidic water occurs naturally through the oxidation of subsurface sulfidic bodies. Therefore, mining is not necessarily the cause of the characteristic low pH and high concentration of metals found in the river. This hypothesis is strongly supported by the sedimentary record of the ancient terrace deposits along the Tinto basin, which largely predate the oldest mining activity in the area [47,48]. The oldest terrace, containing finely laminated, as well as massive ironstones, has been dated 2.1 Ma [48]. Furthermore, the gossan deposits in the area, representing the remaining materials of the *in situ* oxidation of the massive and stockwork sulfide deposits, have been dated as older than 6 Ma [59].

Considering the geomicrobiological characteristics of the Tinto ecosystem, we postulate that the river is predominantly under the control of iron [9]. Iron is the main product of bioleaching of pyrite and iron bearing minerals, like chalcopyrite, both present in high concentrations in the IPB. The activity of iron oxidizing microorganisms is responsible for both the solubilization of sulfidic minerals and the corresponding high concentrations of iron, sulfate and protons detected in the water column of the river.

## 4. Iron World

Iron has diverse properties of ecological interest that make the Tinto ecosystem an interesting focus for astrobiological studies. Iron is not only a source of energy as a chemolithotrophic electron donor in its reduced form (Fe^2+^), but can also be used as an electron acceptor for anaerobic respiration in its oxidized form (Fe^3+^). As mentioned above, ferric iron is responsible for controlling the pH of the ecosystem. Although the reaction is reversible, dilution by tributaries is stronger than evaporation, so an important part of the iron remains precipitated along the course of the river, giving rise to iron bioformations. Accordingly, the concentration of soluble iron decreases gradually from the origin to the mouth of the river. Furthermore, soluble ferric iron readily absorbs harmful UV radiation, protecting the organisms growing in its waters [60,61].

This iron-controlled scenario seems reasonable for the chemolithotrophic prokaryotes thriving in the Tinto ecosystem. However, given the high level of eukaryotic diversity detected in the Tinto basin [18,19] and the fact that most of the primary production of the system derives from the activity of eukaryotic photosynthetic protists, what is the advantage, if any, for eukaryotes to develop in an extreme acidic environment with high concentrations of toxic heavy metals?

A possible answer to this question may be linked to the limited availability of iron in a neutral world. Although iron is an extremely important element for life [4,62], it is a limiting factor for growth at neutral pH [63,64]. Organisms have developed very specific elaborate mechanisms to sequester iron anywhere they can find it [65,66]. Why is this so, when iron is one of the most abundant elements on Earth [4]? In an oxidizing atmosphere at neutral pH, soluble ferrous iron is rapidly oxidized into insoluble compounds, which are incorporated into anaerobic sediments, where sulfate reducing microorganisms may further transform them into pyrite, an even less reactive iron mineral at neutral pH. The geological recycling of these sediments and the microbiology associated with the iron cycle are the only ways to reintroduce this critical element into the biosphere. The possible advantage for the eukaryotes thriving in the extreme conditions of Río Tinto is an unlimited iron supply provided by the chemolithotrophs growing in the rich iron sulfides of the IPB [9,67]. The availability of iron and other heavy metals is considered so important for life that a model for an anoxic Proterozoic ocean deprived of iron and other heavy metals as a consequence of intense sulfate reducing activity has been proposed [68].

## 5. Subsurface Geomicrobiology of the Iberian Pyrite Belt

From the results discussed so far, it is clear that the main characteristics of the Tinto basin are not the product of mining contamination, but a consequence of the existence of an underground bioreactor, in which the massive sulfidic minerals of the Iberian Pyrite Belt are the main energy source, feeding the river with the products of the metabolic reactions occurring in the subsurface. The MARTE (Mars Astrobiology Research and Technology Experiment) (2003–2006) and IPBSL (Iberian Pyrite Belt Subsurface Life Detection) (2011–2015) drilling projects were designed to test this hypothesis by intersecting this underground reactor to provide evidence of subsurface microbial activities.

The main goal of the MARTE project (Mars Astrobiology Research and Technology Experiment), a collaborative effort between NASA and the Centro de Astrobiología, was the search for subsurface microbial activity associated with the IPB. The selected drilling site was Peña de Hierro on the north flank of the Río Tinto anticline. The hydrothermal activity in the area is recorded as complex-massive sulfide lenses or stockwork veins of pyrite and quartz, which occur at the upper part of the IPB volcanic sequence [22]. Three boreholes, BH1, BH4 and BH8, were continuously cored by rotary diamond-bit drilling, producing 60-mm diameter cores protected by a plastic liner. Water, with NaBr as a chemical tracer for controlling contamination, was used as the drilling fluid to refrigerate the bit. Upon retrieval, cores were flushed with N_2_, sealed and transported to a nearby laboratory for geomicrobiological analysis. Samples were prepared aseptically in anaerobic conditions.

After drilling, wells were cased with PVC tubes set in clean gravel packing. Underground sampling for water and gas aquifer analysis was done by the installation of multilevel diffusion samplers (MLDS) at different depths. Ion and metal concentrations and dissolved gases were determined by ion and gas chromatography [69,70].

**Figure 3:**
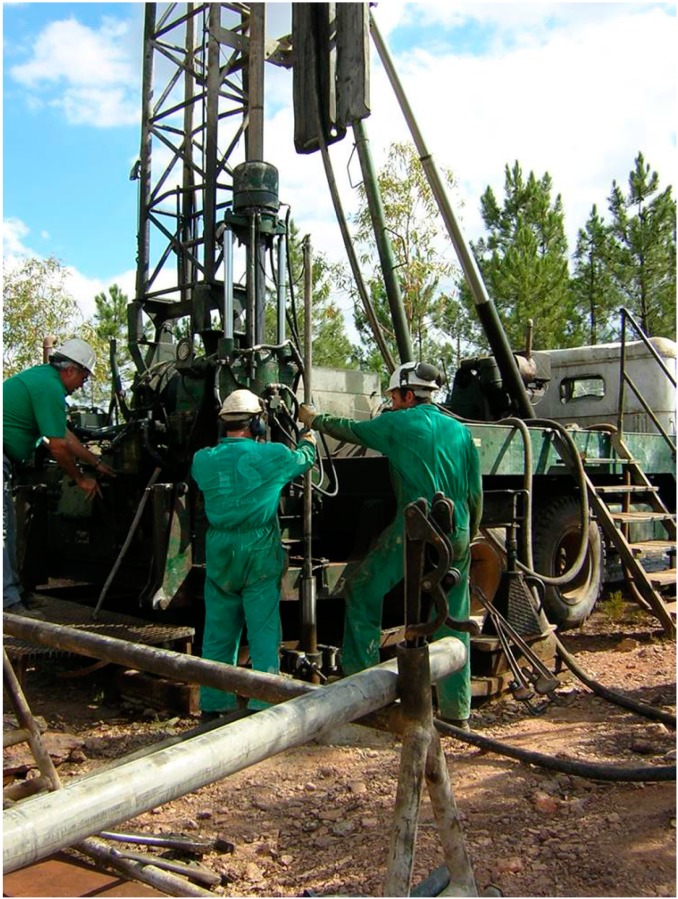
MARTE (Mars Astrobiology Research and Technology Experiment) project, borehole (BH) BH4 drilling site.

The groundwater entering the ore body at Peña de Hierro was characterized by analyzing springs upslope. The water from these springs is aerobic, with neutral pH and low ionic strength. The environment within the ore body was sampled by drilling boreholes BH4 and BH8 (Figure 3). Both wells reached a depth of 165 m. The water table was encountered nearly 90 m below the surface.

Rock leachate analyses were performed to detect drilling contamination and to estimate resources available to microorganisms from the solid phase. Sulfate was abundant and a good indicator of the degree of oxidation of the sulfides. Nitrite and nitrate were present in many samples. Both ferrous and ferric iron could be leached from powdered ore samples, indicating the existence of an operative iron cycle [69,70].

Borehole fluids from the MLDS were analyzed as a proxy for formation fluids. The measured pH was *ca*. 3.5 and remained acidic for two sampling years after drilling. The dissolved ferric-to-ferrous iron ratio varied along the wells ranging from 0.3 to 4.3. Sulfate concentrations were constant and lower than in rock leachates. Surprisingly, dissolved methane was detected in many MLDS samples, indicating active methanogenic activity within the ore body. The dissolved H_2_ concentration averaged 25 ppm, except in the zone within the massive pyrites, just below the water table, where concentrations between 100 ppm and 1000 ppm were detected [69,70].

Microorganisms were detected in different uncontaminated samples using both culture-dependent and culture-independent methods. Aerobic chemolithoautotrophs, mainly pyrite and iron oxidizers, anaerobic thiosulfate oxidizers using nitrate as the electron acceptor, sulfate reducers (SRB) and methanogens were detected in enrichment cultures from core samples at different depths. Samples from both boreholes were analyzed with the microsensor, LDChip200, an antibody microarray containing 200 antibodies with different and complementary specificity, and an oligonucleotide hybridization microarray, which gave positive signals for Gram-positive bacteria, sulfur and metal oxidizers, SRBs, as well as methanogens. Hydrogenotrophic and denitrifying bacteria were also identified by 16SrRNA cloning and sequencing. Using fluorescence *in situ* hybridization (CARD-FISH), it was possible to prove the presence of active microorganisms in different uncontaminated samples [70].

The environment down-gradient from the ore body was sampled by drilling borehole BH1. Sulfate and iron concentrations were lower in the leachates from BH1 shales than those from BH4 and BH8 pyrites, while dissolved sulfate in groundwater was in much higher concentrations than in groundwater from BH4 and BH8, indicating that these waters had experienced more interaction with the sulfides of the IPB. Dissolved H_2_ concentrations were lower than in BH4 and BH8, but still sufficient to make it available as a microbial electron donor. Methane concentrations were several orders of magnitude higher than at BH4 and BH8. Enrichment cultures showed mainly sulfate reducing and methanogenic activities along this borehole [69,70].

**Figure 4:**
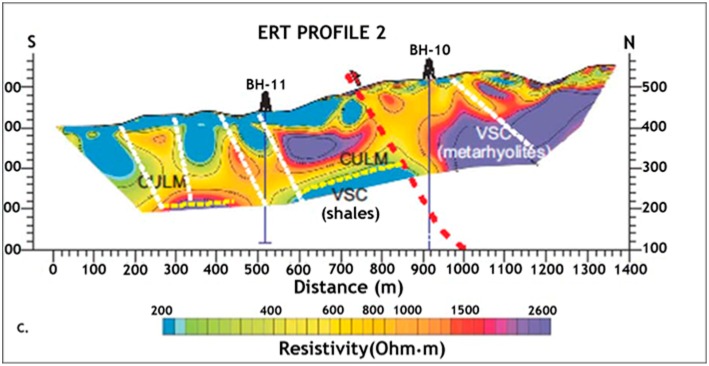
Electric resistivity tomography profile of Peña de Hierro showing the location of the selected drilling sites.

To further investigate the characteristics of the subsurface geomicrobiology of the IPB, researchers at the Centro de Astrobiología applied for an ERC project, which was granted in 2011 and which is currently being carried out (Iberian Pyrite Belt Subsurface Life Detection, IPBSL). Electric resistivity tomography (ERT) (Figure 4) and time-domain electromagnetic sounding (TDEM) were used to detect the most probable subsurface areas hosting microbial activity in deep regions of the IPB. After this geophysical information was analyzed, two wells, BH10 and BH11, with depths of 620 and 340 meters, respectively, were drilled [57].

The IPBSL drilling was performed in conditions similar to those described for the MARTE project. In addition to the geological core login in the drilling site, selected samples were obtained for mineralogical (XRD), elemental analysis (ICP-MS) and stable isotopic and petrographic analyses. The mineralogical results showed the presence of pyrite and its alteration products, such as hematite and magnesite, in both boreholes. The elemental analysis of the leachates from the core samples showed the presence of iron and other metals at different depths. The stable isotopic analysis of pyrites showed ^34^S fractionation at different depths, which is a clear indication of sulfate reducing activities along the borehole. Furthermore, fractionation in ^13^C was observed in samples from both boreholes, which is also a clear biosignature of microbial activity at these depths.

Rock leachates were analyzed by ion chromatography to determine the concentration of water soluble anions. The chromatograms showed the presence of reduced organic anions, like acetate, and oxidized inorganic inions, such as nitrate and sulfate. Protein and sugar content were also detected at different depths, indicating the presence of extant or recent microbiological activities. H_2_, CO_2_ and CH_4_ were detected by gas chromatography of different core samples from both boreholes. Samples along the BH10 borehole were analyzed with the immunosensor, LDCChip450, an antibody microarray containing, in this case, 450 antibodies. Positive signals were detected with specific antibodies against methanogenic Archaea and SRB, which agree with the results obtained by enrichment cultures. DNA and RNA have been successfully extracted from different BH10 and BH11 samples. Most of them rendered positive PCR amplifications of bacterial and archaeal 16S rRNA gene, which are currently under analysis by cloning and massive sequencing. Samples from the two boreholes are being analyzed by fluorescence *in situ* hybridization analysis (CARD-FISH). The results obtained so far showed positive hybridizations signals for both Bacteria and Archaea at different depths. The observed microorganisms appeared in colonies attached to mineral particles in most samples. Further hybridizations with probes selected or designed after identification of putative organisms along the boreholes are under development.

Anaerobic enrichment cultures were prepared in an anaerobic chamber using mineral salt medium with the addition of different electron donors and electron acceptors. The following activities have been detected unambiguously after more than one year of incubation using samples from both boreholes: methanogens, methanotrophs, sulfate reducers, iron oxidizers, iron reducers, denitrifiers and acetogens. From all of the available data, different hot spots have been identified. The detection of hot spots is required for the selection of samples for metagenomic and retro-transcriptomic analyses, which is under way.

The results obtained so far in both drilling projects clearly show that as groundwater enters the volcanogenic-hosted massive sulfide system of the IPB, abiotic and biological processes are activated. Electron donors available for microbial metabolism include ferrous iron, metal sulfides and H_2_. Identified electron acceptors include nitrate, sulfate, ferric iron and CO_2_. These compounds support a community of different microbial metabolisms. In contrast to conventional ARD models, oxidants to drive the system are supplied by the rock matrix. Only mobilization of these sources by ground water is required to promote microbial metabolisms. These observations confirmed the hypothesis that microorganisms are active in the subsurface of the IPB and are responsible for the characteristic extreme conditions detected in the Tinto basin.

## 6. Rio Tinto as a Mars Terrestrial Analog

**Figure 5:**
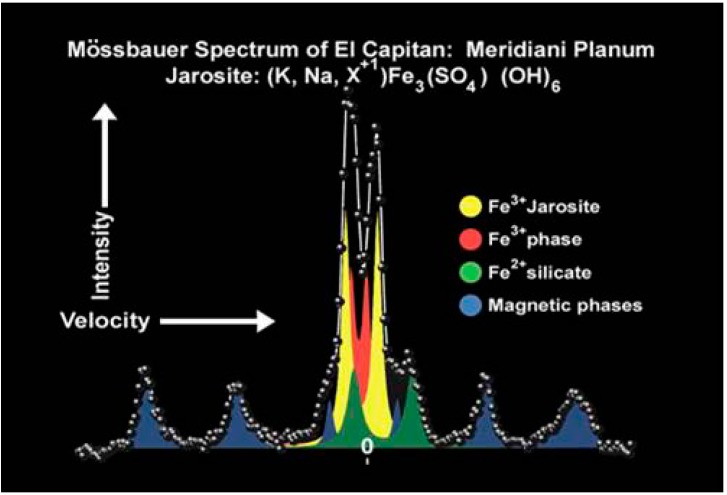
Mössbauer spectrum of jarosite at Meridiani Planum (Courtesy of NASA/JPL-Caltech).

The discovery of some Noachian layered sulfate minerals at different Mars locations suggest a past aqueous, acidic, sulfate-rich environment [71,72,73,74,75] that might have originated from the weathering of sulfide-rich minerals [76,77]. In 2004, the NASA rover, Opportunity, from the Mars Exploration Rovers (MER) mission began its exploration on the Martian surface at Meridiani Planum. The scientific motivation for this selection was the identification by orbital observation of regionally distributed hematite, inferred to have formed under aqueous conditions on the early Martian surface [78,79]. Several hypothesis for hematite deposition have been proposed [78,80], but the observations made by the rover, Opportunity, decisively tipped the balance towards models that invoke pervasive chemical weathering of basalts and subsequent formation of hematite-rich spherules within sulfate rich sediments [71,81,82]. Jarosite, a ferric iron sulfate-hydroxide mineral identified by Mössbauer spectroscopy (Figure 5) [72], placed a particular constraint on the paleo-environmental interpretation of Meridiani outcrop rocks, as this mineral is considered to precipitate under acidic conditions [83].

As has been described previously, jarosite, goethite and hematite are iron minerals that can be found in the Tinto basin as a result of the microbial metabolism of chemolithotrophic microorganisms thriving in the high concentration of iron sulfides of the IPB. Río Tinto as a geochemical and mineralogical terrestrial Mars analogue provides an interesting perspective for the interpretation of Martian data for two reasons. First, both iron oxides and ferric sulfates are generated at Río Tinto under well-characterized physico-chemical and biological conditions [20,36,48,50]. Second, the modern drainage, where depositional processes can be observed in action, is complemented by a historical record of deposition preserved as diagenetically stabilized sedimentary rock in terraces at different levels above the river [47,48]. The combination of ancient and modern deposits facilitates comparison with Meridiani Planum and other iron minerals regions, like Valles Marineris, Mawrth Vallis and Syrtis Major [72,73,74,75], where depositional and diagenetic processes must be inferred from ancient sedimentary rocks [84].

The mineralogical composition and sedimentary geomicrobiology reported for the Tinto basin are of use in addressing several issues of interest in Mars exploration: (i) What biological, chemical and physical processes left an interpretable record in Río Tinto rocks? (ii) How did different processes modify the initial mineralogical and chemical composition of iron-bearing precipitates, and what are the consequences of these processes for the retention of environmental or biological proxy records in Río Tinto rocks? (iii) How much of the informative proxy record might be captured using the suites of instruments used in ongoing Mars exploration missions (Mars Exploration Rovers (MER), Mars Express (MEX), Mars Reconnaissance Orbiter (MOR), Mars Science Laboratory (MSL)) or planned future missions [48,50].

The Río Tinto and Meridiani depositional systems have both similarities and differences. The most obvious difference concerns physical setting. Whereas the Meridiani rocks accumulated via eolic and aqueous processes in an arid environment [85], Río Tinto sediments formed in seasonally arid stream beds. Moreover, the Tinto basin precipitates owe their genesis to the oxidation of hydrothermally emplaced pyrite ores. While this process has also been proposed as a source of sulfuric acid in Mars, it is not the only possible source of acidic fluids that interact with the basaltic rocks in the Meridiani region [84].

The Río Tinto Mössbauer spectra are very similar to those from Meridiani [48] and provide a useful geochemical and mineralogical analog for the processes at play when Meridian rocks were formed. Río Tinto rocks also document the effects of diagenesis, making them doubly useful when compared to Meridiani sediments. Like Río Tinto, Martian outcrop rocks on the Meridiani plain contain hematite, but unlike the Río Tinto terraces, Meridiani outcrops remain sulfate-rich, including ferric sulfates that do not persist into the rock record at Río Tinto. This suggests that mineral formation and diagenesis occurred on Mars under extremely limited water conditions [48,80,85].

**Figure 6:**
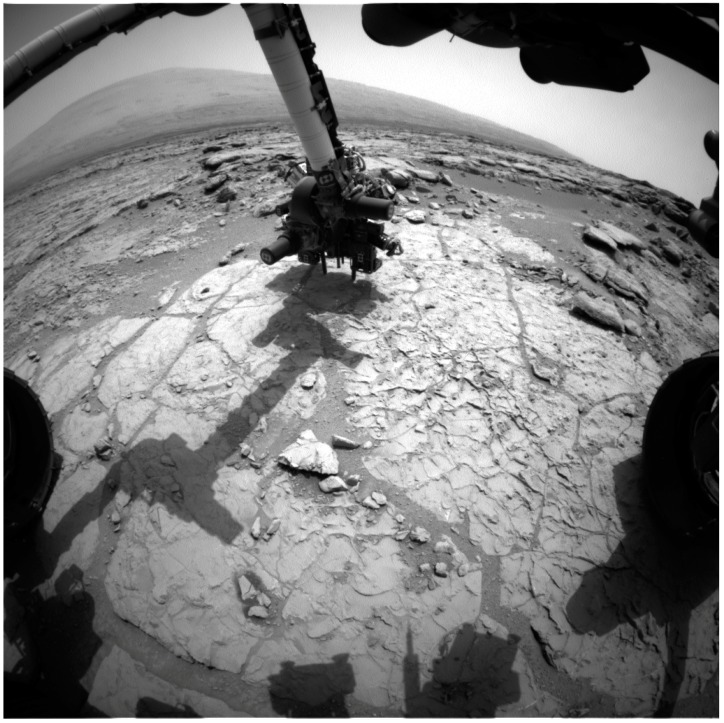
Suite of instruments in Curiosity’s mobile arm (Courtesy NASA/JPL-Caltech).

Diverse microorganisms thrive in acidic and strongly oxidizing environments, which, from an astrobiological perspective, are inferred to be at least broadly similar to those at Meridiani and other Mars iron-rich regions, at the time when their sulfate-rich sedimentary successions were deposited [73,74,75]. Thus, Río Tinto has helped frame some of the biological expectations of Mars exploration. The Río Tinto sedimentary deposits record aspects of the physical, chemical and biological environment of the regional ecosystem, and these indicators persist through diagenesis to provide a geochemical and geomicrobiological chronicle of the Río Tinto processes through time. By inference, Martian outcrops carry a similar potential to preserve a record of the environment and life (if it ever existed or currently exists). To improve Mars characterization, the rover, Curiosity, from the Mars Science Laboratory (MSL) mission (Figure 6) has an XRD for mineral identification and a mass spectrometer to obtain isotopic data.

**Figure 7:**
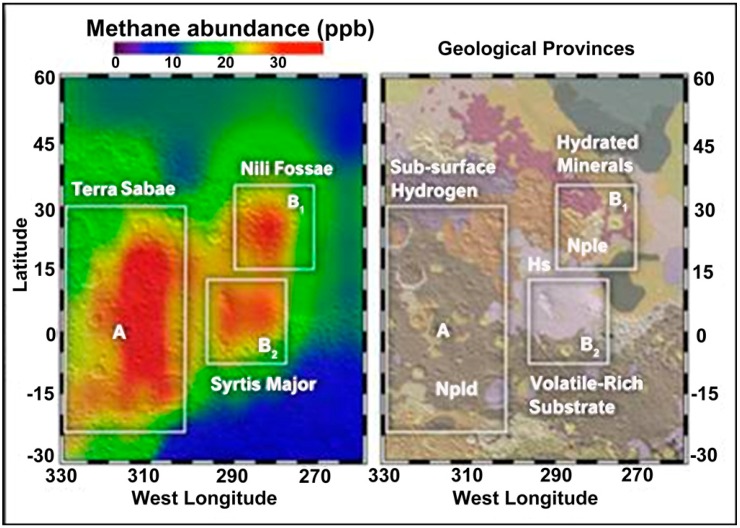
Methane detection on Mars [81] (Courtesy of NASA/Goddard).

The results, to date, in the characterization of the IPB suggest that these Martian systems could support subsurface life, even if surface conditions preclude it. We have found that subsurface microbial metabolism coupled with sulfide weathering can produce large amounts of methane, which has been proposed as an atmospheric indicator of extant life on Mars (Figure 7) [86,87].

Although methane can be generated abiotically, more than 80% of Earth’s methane is biologically produced as a final product of the degradation of organic matter by methanogenic Archaea. Methanogens, with few exceptions [88,89], are generally found in habitats that share two physicochemical properties: reduced redox potentials and circumneutral pH. These conditions are the opposite of the extreme acidic and oxidative conditions existing in Rio Tinto.

After the detection of methane in the Martian atmosphere [86], a systematic survey for methanogenic activity was initiated in the anoxic sediments of the Tinto basin [90]. The first site where methane production was detected was Campo de Galdierias, in the origin area of the Tinto basin. Sediments from this site showed specific positions with negative redox potential, while in the surrounding sediments, the redox potential values were highly positive and similar to those measured in the water column of the river. Microcosms were established using reduced sediments from this site spiked with different methanogenic substrates. In all cases, the production of methane was associated with a decrease in redox potentials to negative values and an increase of pH. The highest methane production was observed in microcosms spiked with methanol [90].

A second site, JL Dam, was selected to have access to deeper and more reliable sediments. Cores from this site showed well-defined black bands with negative reduced redox potentials and higher pH values than the adjacent reddish-brown sediments. Total DNA from these black bands was extracted, amplified, and sequences corresponding to *Methanosaeta concilii* were retrieved [90]. To further explore the methanogenic diversity of the cores, enrichment cultures were designed using different substrates. The highest CH_4_ production occurred in the presence of a mixture of reduced organic compounds. Only *M. concilii* was detected in this microcosm, suggesting that this was the predominant methanogen in environments exposed to organic substrates. *Methanobacterium bryantii* and *Methanosarcina barkeri* were identified in cultures enriched only with H_2_ or methanol, respectively [90].

As mentioned, the bulk environmental conditions at Rio Tinto are far from the conditions required to develop methanogenic Archaea, but this apparent contradiction can be resolved at the microscopic level. The generation of micro-niches might facilitate the growth of microorganisms with different requirements from those found in the harsh bulk conditions existing in the environment. These micro-niches could be easily generated in a semi-solid matrix, such as sediments, or in a solid matrix within a subsurface rock.

The presence of methanogens in an environment controlled by oxidized iron and sulfate has important implications for the characterization of Martian methane [86,87]. The argument that the environmental conditions on Mars are not suitable for methanogenesis could be challenged by the methane production observed in the sediments of Río Tinto or the subsurface of the IPB. Considering the short life span of atmospheric methane on Mars [87], there is a possibility that extant methanogens are currently active on the subsurface of the red planet. As mentioned, the Curiosity rover, currently exploring crater Gale, is equipped with a mass spectrometer capable of measuring the carbon fractionation signal of Martian methane, which might allow its biological or abiotic origin to be clarified.

Although the history of iron in the Earth’s biosphere is still an open question, we would like to suggest that the Tinto ecosystem, as well as other iron-rich acidic environments, are relics of an ancient (Archaean) iron world [9], which is probably operating in other planetary systems, such as Mars [48,50,58]. Obviously the actual conditions in which the Tinto ecosystem operates are different from the ones that may have prevailed during the Archaean or might prevail on Mars, but the properties of the microorganisms isolated and characterized so far in this environment allow us to extrapolate their performance in these systems.

**Figure 8:**
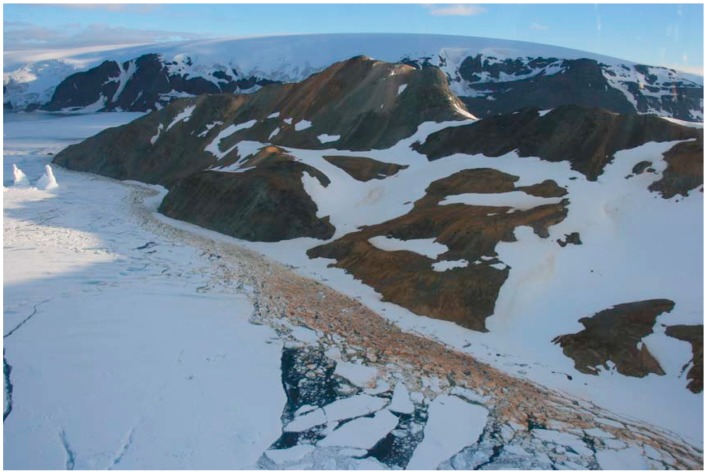
Iron bioformations along the coastline of Cardozo Cove at King George Island [110].

Liquid water seems to be an absolute requirement for life. As indicated, liquid water is abundant in the Tinto basin, both on the surface and underground. Conversely, due to environmental constraints, water appears only in solid or vapor phases on the current Mars surface [91]. Climatic studies of the early atmospheric evolution of Mars [92] indicate that during the Noachian, the atmospheric pressure was high enough to sustain substantial amounts of liquid water on its surface, explaining the above mentioned water-related features. Although we have the orbital technology to reveal the possible existence of liquid water on the subsurface of Mars, so far, there is only indirect evidence of widespread subterranean ice [93], its quantification in the polar water-ice [94] and the characterization of brines from the polar region [95]. However, images from Mars, as well as spectral data provided by different instruments in orbit and on the surface of the planet support the existence of distinctive episodes of water on Mars’ surface in the past [71,96,97,98,99,100,101,102,103]. Although there is only a remote possibility that the Martian iron mineral formations are the product of chemolithoautotrophy, the microbial diversity found in the Tinto basin, with metabolisms compatible with the conditions prevailing on Mars, suggest that microorganisms may have grown or are still growing in places where mineral and water converge. Obviously, Río Tinto is not the only acidic environment of astrobiological interest. In recent years, diverse extreme acidic environments have been identified as terrestrial analogues of Mars, including the seasonally dry acidic lakes of Kalgoorly in Australia [104,105], the cold acid drainage systems in the Canadian Artic [106,107,108,109] and the ARD in King George Island in Antarctica (Figure 8) [110]. Most of these environments have been analyzed from a geological and mineralogical point of view. A thorough geomicrobiological characterization of these sites and others to be explored will eventually complement the information already obtained in the IPB. From a Martian perspective, it would be very useful to take into consideration the possible existence of micro-niches by designing a drilling mission to gather information on the existence of redox gradients of possible use by chemolithotrophic microorganisms.

## 7. Conclusions

Forty years ago, the Viking mission, considered the first astrobiological mission devoted to the search for signs of life on Mars, concluded that life had little chance of developing there due to the extreme conditions detected on its surface. Since then, important advances in microbiology, especially in the characterization of extreme environments, have challenged this pessimistic point of view. Acidophiles are of special interest, because they form the only known natural extreme environment generated by the metabolic activity of chemolithoautotrophic microorganisms. The characterization of the Río Tinto basin, an extreme acidic environment, has addressed some basic issues, including the origin of the extreme conditions of the habitat, the identification and isolation of the microorganisms responsible for these conditions and the existence of micro-niches in the sediments and the subsurface that facilitate the development of microorganisms with requirements incompatible with the bulk conditions existing in the environment. The discovery of some Noachian iron lithological units on Mars, similar to those produced biologically in the Tinto basin, gave Río Tinto the status of a geochemical and mineralogical terrestrial analogue that enables us to better understand those geomicrobiological processes that may have driven the generation of iron oxides and sulfates on Mars.
